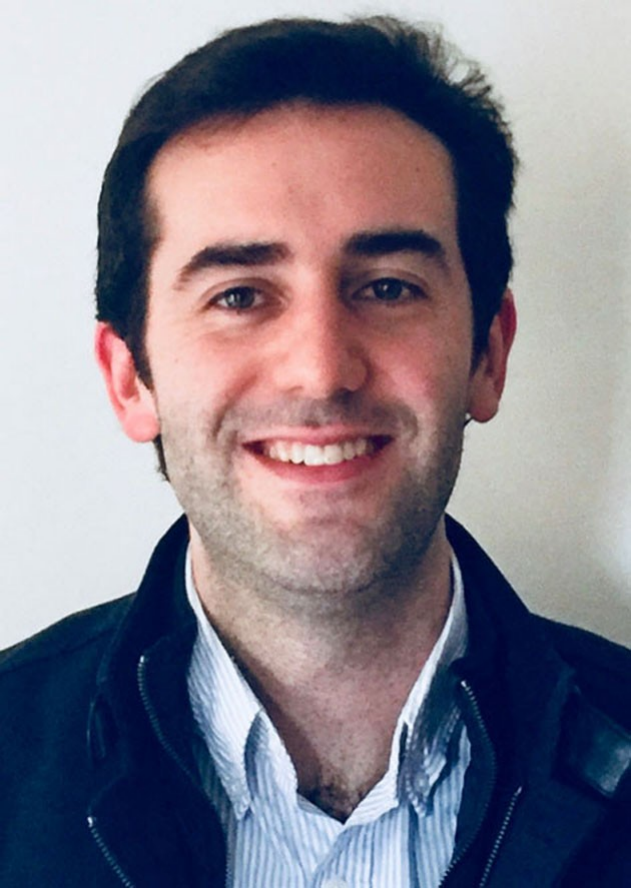# Trends and Innovations in Biosensors for COVID‐19 Mass Testing

**DOI:** 10.1002/cbic.202000250

**Published:** 2020-05-27

**Authors:** Ibon Santiago

**Affiliations:** ^1^ Physics Department Technical University of Munich Am Coulombwall 4a/II 85748 Garching b. München Germany

**Keywords:** COVID-19, CRISPR diagnostics, immunoassays, isothermal amplification, molecular diagnostics

## Abstract

Fast and widespread diagnosis is crucial to fighting against the outbreak of COVID‐19. This work surveys the landscape of available and emerging biosensor technologies for COVID‐19 testing. Molecular diagnostic assays based on quantitative reverse transcription polymerase chain reaction are used in most clinical laboratories. However, the COVID‐19 pandemic has overwhelmed testing capacity and motivated the development of fast point‐of‐care tests and the adoption of isothermal DNA amplification. Antigenic and serological rapid tests based on lateral‐flow immunoassays suffer from low sensitivity. Advanced digital systems enhance performance at the expense of speed and the need for large equipment. Emerging technologies, including CRISPR gene‐editing tools, benefit from high sensitivity and specificity of molecular diagnostics and the easy use of lateral‐flow assays. DNA sequencing and sample pooling strategies are highlighted to bring out the full capacity of the available biosensor technologies and accelerate mass testing.

## Graphical abstract

**Biosensors for COVID‐19 testing**. The landscape of molecular diagnostics for infectious diseases is evolving rapidly to respond to the COVID‐19 outbreak. The sensitivity, specificity and speed of the tests vary depending on the biomarker (viral RNA, viral protein or antibodies against the virus), sample type (swab or blood) and detection method. Emerging diagnostic tools aim to circumvent roadblocks encountered at each step.

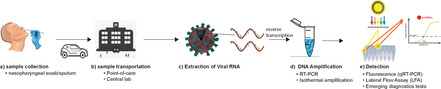

## 1 Introduction

The coronavirus disease 2019 (COVID‐19) is an infectious disease caused by the severe acute respiratory syndrome coronavirus 2 (SARS‐CoV‐2).^1^ The outbreak was declared by the World Health Organisation (WHO) to be a public health emergency of international concern (PHEIC).^2^ Since December 2019, when it was first reported in Wuhan (China), COVID‐19 has put more than a billion people in quarantine and has brought the economies of many countries to a halt. Governments responded with diverse strategies, informed on epidemiological models.^3^ However, a lack of information on the real number of infected cases and the actual case fatality rate (CFR) has led to large uncertainties in quantifying and predicting the extent of this pandemic.^4^

To obtain this key information, reliable diagnostics of COVID‐19 for surveillance and tracking of new infections become essential. The development of tests for SARS took about six months.^5^ With COVID‐19, the short time it took from the detection of the first case (Nov. 2019) to the sequencing of the viral genome by Chinese scientists (11 Jan. 2020)^1^ and the development of the first molecular assay (13 Jan. 2020) has been remarkable.^6^ But testing in many countries is still minimal or rationalised, as the testing capacity is overwhelmed by the extent of the outbreak. This has led to significant variations in testing numbers per capita across countries.^7^ Insufficient statistically well‐designed testing prevents the accurate determination of the number of cases of infection and leads to a more than likely overestimate of the CFR.^8^ CFR estimates are around 1.38 %, higher than the 0.1 % CFR of seasonal influenza.^9^

Unreported infections (mostly asymptomatic or mild cases) increased the number of cases around the world^11^ and expanded the pool of individuals who need screening. Testing and contact tracing have become essential in the process of loosening lockdowns. Countries with widespread testing and surveillance strategies like South Korea and Germany have been able to contain the spread.^12^

**Figure 1:**
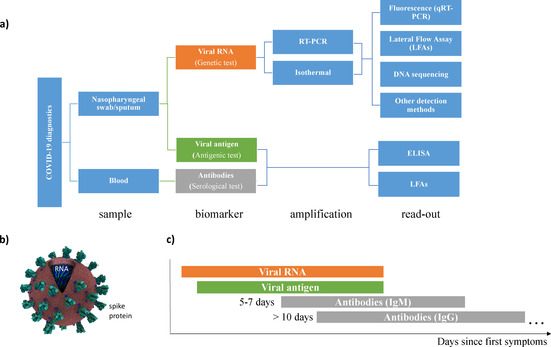
a) Scheme of Covid‐19 diagnostics tools and workflow based on sample type, biomarker (genetic, antigenic or serological), signal amplification and detection method. b) Representation of SARS‐CoV‐2 (adapted from RCSB PDB, credit: Maria Vogt). c) The suitable time window for different test types. The stage of the disease determines the viral load and antibodies present in the patient.^10^

The focus of this work is how to detect SARS‐CoV‐2 efficiently on a large scale and with high sensitivity. Figure 1 shows the diversity of diagnostic tools targeting biomarkers for COVID‐19 at different stages of the disease. We distinguish three types of tests based on the biomarkers they address: genetic (targeting the viral genome), antigenic (targeting viral proteins) and serological (targeting antibodies against the virus). An in‐depth analysis and comparison of their performance reveal the trade‐offs between testing speed, sensitivity, specificity and ease of use. Technology innovations are in a race to provide high‐performance devices for fast and scalable tests. Isothermal amplification methods, CRISPR‐based diagnostics and next‐generation sequencing adapted for COVID‐19 testing are disrupting the molecular diagnostics landscape. This work identifies bottlenecks hindering widespread testing and presents solutions to make sensitive testing for COVID‐19.

## 2 Genetic Testing

### 2.1 Overview of the genetic testing protocols and innovations

Genetic tests use molecular diagnostic assays to detect the presence of viral RNA specific to SARS‐CoV‐2. National laboratories and companies around the world have developed different primers and probes addressing various regions of the viral genome. For example, the CDC in the USA addresses three targets in the nucleocapsid N gene.^13^ In contrast, the Charité protocol from Germany targets the RNA‐dependent RNA polymerase (RdRP), *E* and *N* genes.^6^

**Figure 2:**
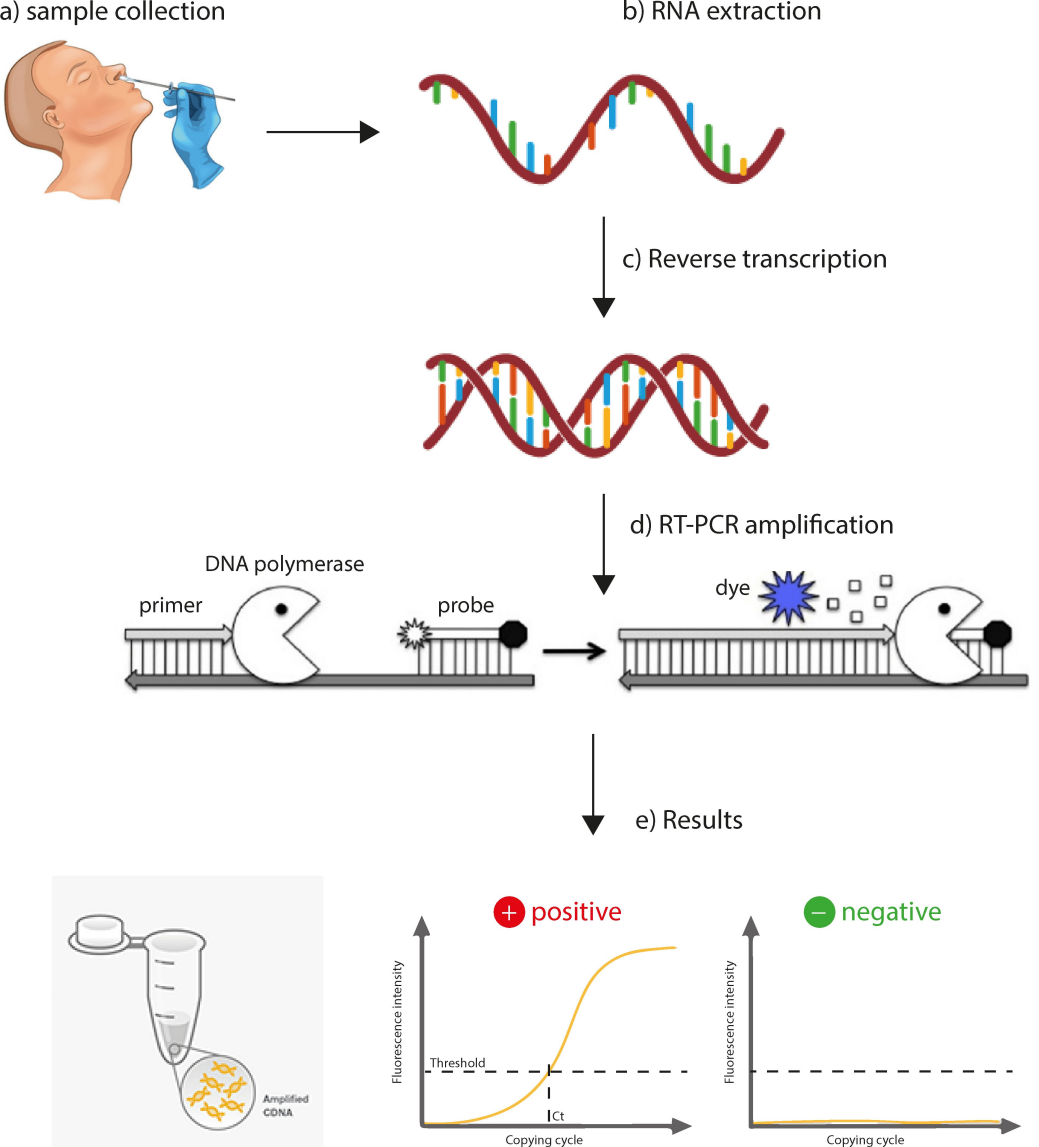
Steps in the qRT‐PCR test: a) Specimen is taken from the nose or throat of individual, b) RNA is extracted and c) is transcribed into complementary DNA (cDNA). d) Once the primers have bound to the DNA, they provide a starting point for the DNA polymerase to help copy it. DNA polymerase then degrades the bound probe, which results in an increased fluorescence signal. e) The fluorescence signal increases as copies of the virus DNA are made. If the fluorescence level crosses a certain threshold, the test result is positive.

Every genetic testing protocol follows analogous steps, including sample preparation, RNA extraction, transcription of RNA into complementary DNA (cDNA), amplification of DNA and read‐out. Figure 2 illustrates the process based on the gold standard test used in most clinical settings, that is, quantitative reverse transcription polymerase chain reaction (qRT‐PCR). The section below identifies bottlenecks in each step and highlights the current research efforts and industrial innovations to address them.

*Sample collection*: A swab is taken from the nose or throat of the potentially infected individual (Figure 2a). There is evidence to suggest that the largest viral load is obtained from nasopharyngeal samples.^14^ Sample collection requires clinical staff and follows a special biosafety procedure. New protocols and technologies are moving towards self‐swab sample collection. Saliva samples also have diagnosis value,^15^ and respiratory virus shedding in exhaled breath^16^ makes breath condensate a potential specimen for diagnosis.^17^

*RNA extraction*: The sample is first inactivated with a lysis buffer. RNA is then isolated and purified (Figure 2b) using fast spin‐columns (e. g., QIAamp Viral RNA Mini Kit by QIAGEN), paramagnetic beads or phenol‐guanidine isothiocyanate (GITC)‐based solutions. A shortage of commercial RNA extraction kits has motivated many laboratories to find in‐house extraction methods: such as Trizol‐based purification^18^ and RNA precipitation with isopropanol.^19^ At least four groups have shown that the RNA extraction step can be omitted, yielding results with minimal change in sensitivity.^20^

*Reverse transcription and amplification*: Reverse transcription is a standard step that uses the enyzme reverse transcriptase to convert RNA into complementary DNA. Amplification is usually performed with PCR. The average processing time for thermal cycling is about 2 hours. Isothermal amplification (DNA replication at constant temperature) is an alternative to PCR that is gaining traction in many point‐of‐care diagnostics devices. Both PCR and isothermal amplification are discussed in the next sections.

*Signal read‐out*: Amplification and read‐out occur simultaneously in qRT‐PCR systems. This is achieved by using fluorescent probes or intercalating dyes, which require bulky, expensive thermal cyclers with embedded fluorescent lamps and detectors. To miniaturise and reduce the cost of the device, alternative read‐out technologies use bead arrays, lateral‐flow assays or electrochemical sensors. Among these innovations, CRISPR‐Cas‐based and DNA sequencing‐based diagnostics stand out, which combine several of the technologies outlined above with innovative amplification and read‐out schemes.

### 2.2 qRT‐PCR as the gold standard COVID‐19 diagnostic test

qRT‐PCR is the primary tool to diagnose COVID‐19. It requires a forward primer to start DNA synthesis, a backward primer and a fluorescent probe, together with reverse transcriptase and DNA polymerase (responsible for DNA replication). In a single or two‐step RT‐PCR, RNA is converted first into complementary DNA (Figure 2c), and then the DNA signal is amplified by a real‐time polymerase chain reaction (a.k.a quantitative PCR). In real‐time PCR, the probe strand (containing two dyes: a reporter and a quencher dye) binds to a specific target sequence to SARS‐CoV‐2 located between the forward and reverse primers (Figure 2d). During the extension phase of the PCR cycle, the polymerase degrades the bound probe, causing the reporter dye to separate from the quencher dye, resulting in an increased fluorescent signal. The fluorescence intensity is monitored at each amplification cycle. This fluorescence signal increases as more copies of DNA are produced, and if it crosses a certain threshold, set above expected background levels, the test result is positive. If the virus was not present in the sample, the fluorescence threshold is not reached, and the test result is then negative (Figure 2e). The cycle threshold (Ct) is the number of PCR cycles required to achieve such a threshold (i. e., exceed the background level). Internal positive (samples known to contain SARS‐CoV‐2 RNA) and negative controls are run in parallel to confirm the validity of the result.

The ideal diagnostic test has both high sensitivity (true positive rate) and specificity (true negative rate). The sensitivity is reported together with the limit of detection (LoD). The LoD sets the lowest concentration of SARS‐CoV‐2 RNA that can be detected by the RT‐PCR test, which is determined by detecting the presence of the viral RNA in at least 95 % of the cases. For COVID‐19 assays, the LoD can reach levels lower than 10 genome copies per reaction (0.5 cp/μL).^21^ However, the sensitivity varies depending on the chosen kits and PCR instrument (Table 1). Viral loads in the upper respiratory tract peak in the first week of symptoms. Failure to detect the virus in infected patients (false negatives) can be due to low sensitivity or other issues, such as laboratories working under pressure, or poor sample collection and preparation. It is yet unknown what types of specimens are optimal for detection with RT‐PCR. A recent study from Wuhan suggests that nasopharyngeal swabs may offer greater consistency than other types of samples.^22^ These tests could also lead to false positives if, for example, specimens are contaminated, or the protocol is not followed appropriately.

**Table 1:** List of COVID‐19 test suppliers, corresponding sensing technologies and reported limit of detection and sensitivities.

Supplier	Sensing technology	Target(s)	Limit of detection	Sample‐to‐answer time	Point of care?
CDC^[a]^	qRT‐PCR	N‐gene	3.2 copies/μL	>2 h	no
Thermo Fisher^[a]^	qRT‐PCR	ORF1ab, N gene, S gene, MS2	10 copies/μL	>2 h	no
Roche^[a]^	qRT‐PCR	ORF1a	9 copies/μL	>2 h	no
PerkinElmer^[a]^	qRT‐PCR	ORF1ab, N Gene	0.025 copies/μL	>2 h	no
Cepheid^[a]^	qRT‐PCR	N‐Gene, E‐Gene	0.25 copies/μL	45 min	yes, GeneXpert Xpress System
BioFire^[a]^	qRT‐PCR	ORF1ab, ORF8	0.33 copies/μL	50 min	yes, FilmArray Systems
Mesa Biotech^[a]^	RT‐PCR+ lateral‐flow technology	N‐Gene	200 copies/reaction	30 min	yes, Accula Dock or the Silaris Dock
Abbott^[a]^	isothermal nucleic acid amplification	RdRp Gene	0.125 copies/μL	5 / 13 min	yes, ID Now platform
Abbott^[a]^	chemiluminescent microparticle immunoassay	IgG	100 % sensitivity	29 min	no, ARCHITECT system
Roche^[a]^	electrochemiluminescence immunoassay	IgG	100 % sensitivity 14 Days post‐PCR confirmation	18 min	no, Elecsys system
Bio‐Rad^[a]^	microplate‐based ELISA test	IgM, IgA, IgG	98 % sensitivity	<44 min	no, EVOLIS system
Sugentech	lateral‐flow assay	IgM, IgG	94 % sensitivity vs. RT‐PCR	10–15 min	yes
Pharmact	lateral‐flow assay	IgM, IgG	70 %–98.6 % sensitivity vs. RT‐PCR	20 min	yes
Quidel^[a]^	lateral‐flow assay	Nucleocapsid protein	80% sensitivity	∼15 min	yes, SOFIA system
Mammoth Biosciences	CRISPR‐based lateral‐flow assays	E‐gene, N‐gene	70–300 copies/μL	∼30 min	yes
Sherlock Biosciences^[a]^	CRISPR‐based lateral‐flow assays	S gene, Orf1ab	10–100 copies/μL	∼60 min	yes

[a] Assays with emergency use authorization (EUA) from the Food and Drug Administration (FDA).

The RT‐PCR test is only indicative of whether the virus is present at the time the test is taken. It neither rules out whether the patient was infected in the past‐and therefore might have developed immunity‐nor that the patient is at an early infection stage and will show symptoms in the future.

### 2.3 Isothermal amplification and point‐of‐care molecular diagnostics

The long processing times and high demand for testing kits and ancillary ingredients needed for sample preparation and processing represent serious bottlenecks for widespread testing. The whole process can provide results within 4 to 6 hours, although some centralised labs might require up to 48 hours from sample to answer. To respond to these challenges some companies are providing their kits and proprietary rapid point‐of‐care molecular diagnostic systems, mostly based on RT‐PCR, which include robotic automation and microfluidic handling of samples (e. g., BioFire and Cepheid).

As an alternative to PCR, the isothermal amplification of nucleic acids allows for extreme amplification of nucleic acids at constant temperatures. It bypasses the thermal cycling process in PCR, thereby shortening the test time and equipment cost significantly. Multiple isothermal amplification technologies have been developed, and some are already used for diagnosis of infectious diseases.^23^ These methods include nucleic acid sequence‐based amplification (NASBA), loop‐mediated isothermal amplification (LAMP), helicase dependent amplification (HDA), rolling circle amplification (RCA), nicking enzyme amplification reaction (NEAR) and strand displacement amplification (SDA). Among them, RT‐LAMP has been adopted for COVID‐19 diagnosis by several groups.^24^

Circumventing the need for thermal cycling means that the use of isothermal amplification decreases the complexity of the diagnostic device and reduces its cost. The amplified DNA can be detected by changes in turbidity, by addition of intercalating dyes or by a pH‐sensitive dye.^25^ This simple read‐out, the fast amplification and the possibility of multiplexing are the main merits of using isothermal amplification over PCR. The low cost and suitability for miniaturization of isothermal amplification methods will encourage a higher take‐up of this technique for point‐of‐care molecular diagnostics.^26^ The main challenges of methods like RT‐LAMP are the difficulty of optimising reaction conditions and designing sequence‐specific primers. But software tools can ease this problem.^27^ The complexity of primer design and their multiplicity might lead to non‐specific amplification. Therefore, a detection method to identify the amplified gene can be integrated to improve the performance. One promising solution involves using the highly specific CRISPR associated proteins Cas.

### 2.4 CRISPR‐based diagnosis

CRISPR‐based lateral‐flow assays are a new addition to the quickly evolving molecular diagnostics landscape. CRISPR is a powerful gene‐editing tool^28^ that has already led to ground‐breaking therapeutic results in clinical trials in the past years. Going beyond its capacity to act as “molecular scissors”, CRISPR and its associated proteins exhibit properties that can be harnessed to detect specific nucleic acids in a sample.^29^

In a race for CRISPR‐based diagnostics, Mammoth Biosciences and Sherlock Biosciences are using CRISPR as a molecular diagnostic tool, rather than as an editing tool, to create fast, cheap and accurate tests to detect infectious diseases, including COVID‐19. Mammoth uses CRISPR technology developed from Jennifer Doudna's laboratory and has created a DNA endonuclease‐targeted CRISPR trans reporter (DETECTR) platform to develop tests that can detect multiple coronavirus strains.^30^ Feng Zhang and colleagues at the Broad Institute have created specific high‐sensitivity enzymatic reporter unlocking (SHERLOCK).

The testing systems work by employing CRISPR nucleases, programmed to find a defined gene sequence. SHERLOCK uses the cleavage and degradation of neighbouring ssRNA by the proteins Cas13 to cleave and activate a fluorescent reporter, whereas DETECTR uses ssDNA and Cas12a. Upon finding the sequence of interest, the nuclease activates a cleavage capability, which generates a fluorescence signal after cleaving a reporter DNA strand present in the sample. The fluorescence signal confirms the sequence has been found.

**Figure 3:**
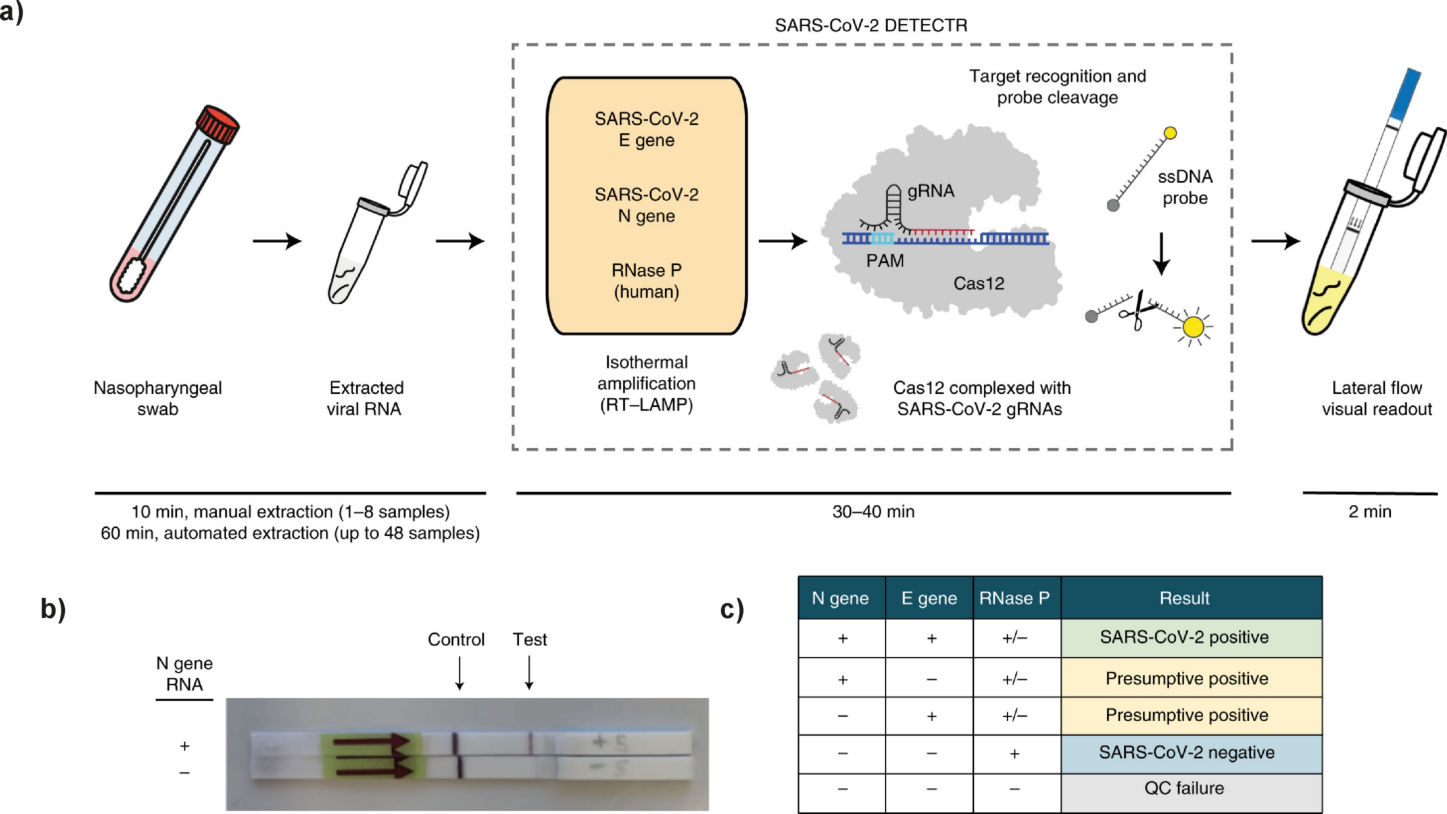
CRISPR‐based diagnosis (DETECTR) by Mammoth Biosciences. a) Schematic of the SARS‐CoV‐2 DETECTR workflow. Conventional RNA extraction is used as an input. It is followed by reverse transcription, loop‐mediated isothermal amplification (LAMP) and Cas12‐based detection of target genes (E, N and RNase P). These are visualised on a lateral‐flow strip. b) Lateral‐flow strip assay read‐out: The line closest to the sample pad is the control line, and the line that appears farthest from the sample pad is the test line. c) Interpretation of results: a positive result requires the detection of at least the two SARS‐CoV‐2 viral gene targets (N gene and E gene). Adapted with permission from ref. [30]'with permission Copyright: 2020, *Springer Nature*

The workflow for both systems after RNA extraction requires pre‐amplification of DNA or RNA and ends with a lateral‐flow assay to reveal the results. As an example, Figure 3 shows the steps involved in DETECTR. The platform first performs reverse transcription and isothermal amplification using loop‐mediated amplification (RT‐LAMP), as an isothermal alternative to RT‐PCR. It is followed by Cas12 detection of predefined coronavirus sequences, after which cleavage of a reporter molecule confirms the presence of the virus. The result is then visualized on a lateral‐flow strip (Figure 3b), together with a control. The full assay can be run in approximately 30 min. DETECTR is able to distinguish SARS‐CoV‐2 with no cross‐reactivity for related coronavirus strains and has a sensitivity comparable to conventional methods, pending FDA approval. Sherlock Biosciences and the Broad Institute have also developed an analogous COVID‐19 specific protocol with FDA approval^31^ and scaled up the capacity by using microfluidic chips that can run thousands of tests simultaneously.^32^

CRISPR diagnosis methods benefit from the high sensitivity of molecular diagnostics, the high selectivity of CRISPR and the fast speed and facile use of lateral‐flow assays. They are adaptable to new targets and offer a potentially rapid and specific read‐out. They can also be run several times on the same sample, decreasing the chance of false negatives. Further, the tests are being designed for the point‐of‐care setting, enabling cheap and widespread screening. Omitting the amplification step would be a significant improvement in this technology.

### 2.5 DNA sequencing‐based diagnosis of COVID‐19

Sequencing of the viral genome helps identify and classify novel strains of coronavirus.^33^ It is also an essential tool to find suitable primers for RT‐PCR diagnostics and guide the search for therapies.^34^ As the virus multiplies and spreads, random mutations accumulate in the genome at an approximate rate of two mutations per month (half the mutation rate of seasonal flu). These mutations can help track the spread of the virus in real‐time and understand its phylogenetics, from which its transmission dynamics can be inferred.^35^

**Figure 4:**
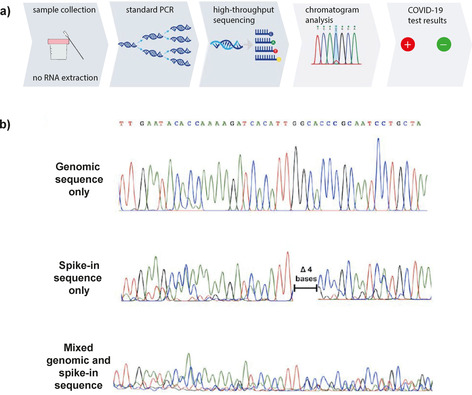
a) Workflow of whole‐genome sequencing for COVID‐19 diagnostics. The sample is collected, and reverse transcription and PCR amplification of a SARS‐CoV‐2 target region are performed without RNA extraction by direct addition to a one‐step RT‐PCR master mix, together with a synthetic spike‐in strand. After co‐amplification of spike‐in and sample on a thermal cycler, the amplified products are sequenced, and the resulting chromatograms are analysed to determine if the sample contains viral RNA. b) A synthetic spike‐in RNA with a 4 bp deletion is designed with sequence homology to the SARS‐CoV‐2 target so that it co‐amplifies with the SARS‐CoV‐2 target. This enables quantification of relative abundances of spike‐in and SARS‐CoV‐2 DNA from a Sanger sequencing chromatogram. Representative Sanger sequencing traces showing pure genomic sequence (top), pure spike‐in sequence (middle), and sequencing from a mixture of genomic and spike‐in sequences (bottom). When both spike‐in and viral genomic sequence are present, the signals are used to estimate their relative abundances. Adapted with permission from ref. ^[37]^. Copyright: 2020, *the authors*.

Apart from surveillance testing, whole‐genome sequencing can provide high‐throughput diagnosis.^36^ Several companies have repurposed sequencers‐including next‐generation sequencers (NGS) and Sanger sequencers‐ for COVID‐19 diagnostics, following a similar workflow shown in Figure 4a. First, they lyse cells from the sample without extracting RNA, thereby saving reagents and reducing the number of steps. Spike‐in RNA is added to co‐amplify with any present viral RNA. The “spike‐in RNA’”‐with a 4 bp deletion‐acts as control and reference for normalisation, to quantify the virus concentration (Figure 4b). A PCR amplification step is performed, followed by NGS or Sanger sequencing. Using this method, BilliontoOne reports capacity to complete about 4000 tests per day with a single Sanger Instrument (30x faster than qPCR).^37^

## 3 Immunoassays (Antigenic And Serological Tests)

### 3.1 Overview of antigenic and serological tests

For scaling up the diagnosis of COVID‐19, rapid‐tests that rely on lateral‐flow assays are an attractive alternative to the qRT‐PCR test. These cellulose‐based devices are less reliable than RT‐PCR tests but can be performed at the point‐of‐care, or in community settings without the need for expensive equipment. Using antibody‐antigen recognition, rapid antigenic tests detect the presence of viral proteins, whereas serological tests target antibodies against SARS‐CoV‐2. It takes at least 5 days to acquire enough viral load to be detectable through antigenic tests and up to 7 days or longer to develop antibodies against the virus (Figure 1c). The stage of the disease is a key parameter determining the choice of test.

**Figure 5:**
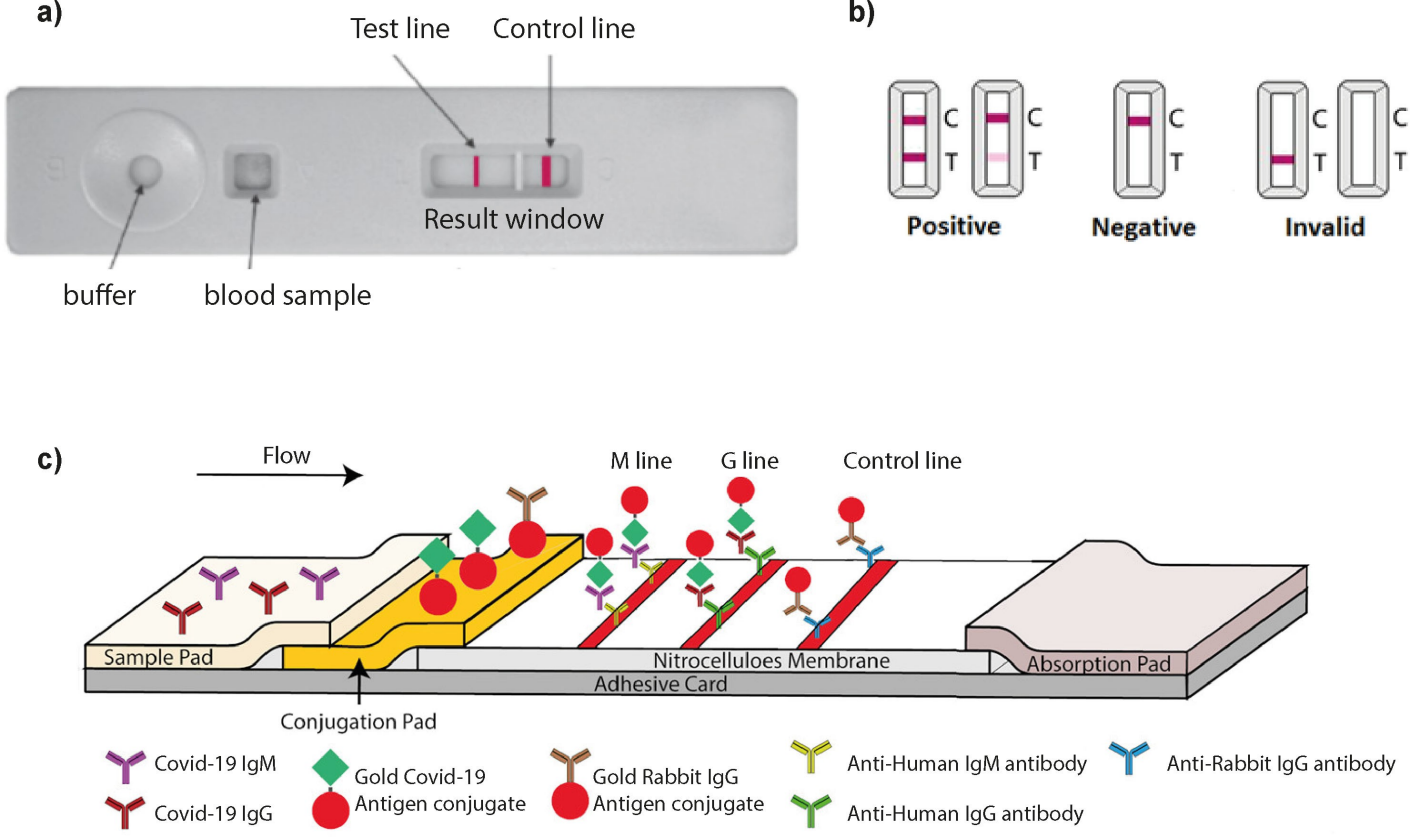
Typical lateral‐flow assay for a serological test. a) Inside the cassette is a strip made of filter paper and nitrocellulose. Typically, a drop of blood is added to the cassette through one hole (sample well), and then a number of drops of buffer are added usually through another hole (buffer well). The buffer carries the sample along the length of the cassette to the results window. b) Interpretation of results. c) A schematic of a COVID‐19 lateral‐flow test. The antibody binds to an antigen conjugated to colloidal gold in the conjugation pad, and the resultant complex is captured on the strip by a band of bound antibodies, forming a visible line (T: test line) in the results window for IgM and IgG. A control line (C) gives information on the integrity of the antibody‐gold conjugate. Adapted with permission from ref. [41]. Copyright: 2020, the authors.

Figure 5 shows a typical lateral‐flow immunoassay, together with the interpretation of its results. Antigenic test strips are coated with antibodies that bind to a viral protein.^38^ Some prototypes use aptamers instead.^39^ The viral proteins in the blood sample bind to the antibodies forming a coloured indicator on the strip. This change of colour is normally induced by using the plasmonic properties of colloidal gold,^40^ which provide a gain in sensitivity without losing the simplicity of lateral‐flow assays.

Serological rapid tests use the same principle as other immunoassays, but instead of detecting viral antigens, the assay detects the presence of antibodies against the virus in the patient sample, namely immunoglobulin G (IgG) and M (IgM). Their concentration increases with time until a plateau is reached for IgG, which can remain for long in the patient after recovery. Therefore, detection of IgG is indicative of past infection and potential immunity. Serology tests can be used to detect current and past exposure to SARS‐CoV‐2 and can be performed in batches in a laboratory or individually at point‐of‐care settings.

Unlike antigenic testing, which can act as a complement to qRT‐PCR, serological testing plays a completely different role in the response to an outbreak and should not be used for clinical decision‐making. Quantifying the number of past infections helps assess the true extent of an outbreak and inform prevention and control strategies. Preliminary seroprevalence studies^42^ point towards a higher prevalence than indicated by the number of confirmed cases. These studies may have overestimated the fraction of those exposed and potentially developed immunity against COVID‐19 in affected populations. As more studies with sensitive and specific tests are done, the precision of the prevalence estimates will improve.

### 3.2 Bottlenecks of immunoassays and emerging technologies

Current rapid tests for influenza suffer from suboptimal sensitivity to rule out disease.^43^ The same challenge is likely to exist for SARS‐CoV‐2 antigenic and serological tests. A problem with immunoassays is that antibodies might cross‐react, and SARS‐CoV‐2 tests could also give a positive result with other types of coronavirus. Nucleocapsid proteins of coronaviruses are highly conserved, especially between SARS‐CoV‐2 and SARS‐CoV.^44^ Assays for SARS‐CoV have the potential to be used to cross‐detect SARS‐CoV‐2 and have been used in the early development of COVID‐19 rapid‐tests, sometimes with suboptimal sensitivity. As many companies around the world race to produce rapid tests, probing the accuracy of such tests will require independent trials with hundreds of known SARS‐CoV‐2 infected cases before deployment in community settings.

There is a trade‐off between sensitivity and speed in rapid tests. Although point‐of‐care lateral‐flow immunoassays have been developed to diagnose COVID‐19 within 15 minutes (Table 1), the concentration of the analyte usually needs to be higher than 10 copies/μL. This means that most of these tests may only work in symptomatic individuals, although some tests might be capable of detecting infection at early stages. Some countries have rushed into large scale deployment of rapid tests, finding that the clinical sensitivities are lower than 30 %. The performance of rapid tests provided by manufacturers might vary from results in a routine testing laboratory. This is why clinical validation of rapid tests should be compared with a gold standard test in a large number target population before using them as a stand‐alone diagnostic test.^45^ Point‐of‐care molecular diagnostic assays can be compared against the RT‐PCR standard, but the validation of antibody tests is more challenging. Viral proteins required for the assays are hard to procure, as well as patient blood samples with which to validate the assays. The Foundation for Innovative New Diagnostics (FIND), in partnership with WHO, is conducting independent evaluations of molecular diagnostics and immunoassays to help generate performance data.^46^ An evaluation of antibody testing for SARS‐CoV‐2 using enzyme‐linked immunosorbent assay (ELISA) and lateral‐flow immunoassays in the UK concluded that many commercial antibody rapid tests were not suitable for clinical use.^47^ Even if the more accurate ELISA tests are done in centralised labs, it is not yet known whether seroprevalence implies immunity and whether all recovered patients still carry antibodies.

Improving the sensitivity and specificity of immunoassays is the focus of current technological innovations. One solution involves using dyes or nanoparticles to amplify the antibody‐antigen binding signal. A vast array of materials is used as labels to conjugate to the bio‐receptors, with colloidal gold nanoparticles being the most used.^41^ Recent research uses nanoparticles (carbon nanoparticles), luminescent nanoparticles (quantum dots, fluorescent quenching material), liposomes and enzymes.^48^ Labels either generate a signal directly (such as the characteristic red colour of colloidal gold) or require additional steps to produce a signal (such as an enzymatic step).

A digital reader or analyser can significantly improve the sensitivity of the test, for example, ELISA test. Still, it comes at the expense of longer processing times and the need for a fluorescence read‐out. Another trend is to develop fully automated systems for large test throughput (Table 1). One example is the Roche COVID‐19 electrochemiluminescence immunoassay (ECLIA) which is capable of running 300 serology tests/hour depending on the analyser.

## 4 Multi‐sample Pooling for Rapid COVID‐19 RT‐PCR Tests

**Figure 6:**
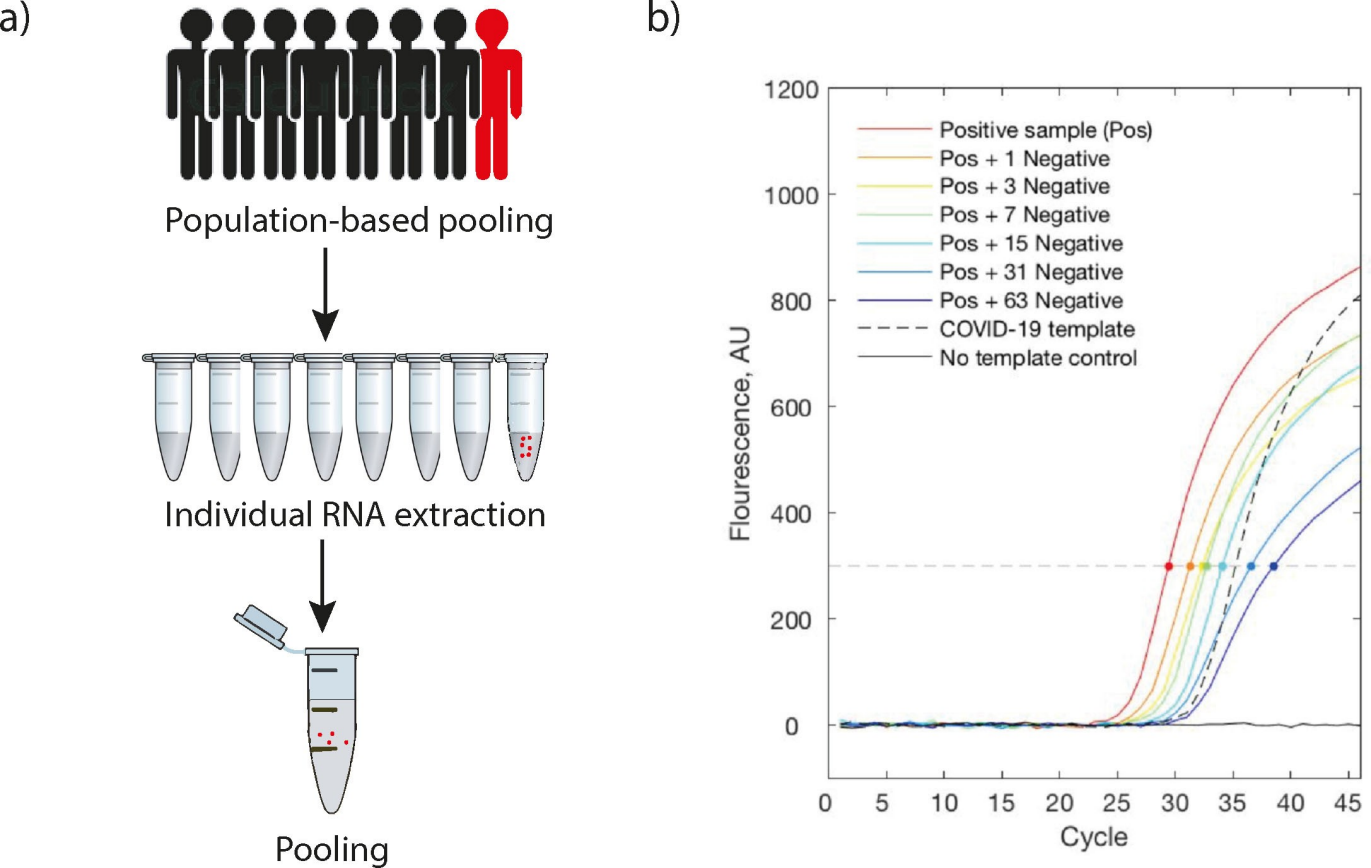
a) Scheme of the pooling strategy: individual samples are collected, RNA is extracted, and up to 64 samples are pooled, out of which one individual is infected (red). b) Representative RT‐qPCR fluorescence curves of a positive sample (Pos) diluted in different numbers of negative samples (red: no dilution, blue: dilution in 63 negative samples). Dots represent the cross point of the fluorescence threshold; extracted with permission from ref. [49a]. Copyright: 2020, the authors.

Researchers have accelerated the RT‐PCR testing rate by taking a pooling approach, enabling the simultaneous testing of dozens of samples.^49^ Preliminary results show that group testing can identify a positive sample among 64 different samples with enough sensitivity (Figure 6). No new technology is needed, but the necessary logistics to implement the pooling strategy, substituting the current individual testing. Representative RT‐PCR scans of pooled samples yielded results below a cycling threshold of 40 (Figure 6b). Such pooling methods, if scaled up appropriately, could lead to mass testing, make better use of current resources and quickly reject negative cases. Sample collection and RNA extraction represent further bottlenecks in testing capacity. By the omission of the RNA extraction step (∼1 h) and adopting a medium‐size pooling strategy (e. g., 5 samples per pool), RT‐PCR testing could be sped up by one order of magnitude.

Universal testing is not a realistic goal for most countries. Instead, taking a representative sample of the population (e. g., through a census) and performing pooled RT‐PCR tests may be a sensible way forward.

## 5 Summary and Outlook

Table 1 shows the leading suppliers of assays adapted for COVID‐19, covering most of the biosensor technologies discussed in this work. They vary in sensitivities (limit of detection) and sample‐to‐answer time. Most commercial COVID‐19 tests that have been granted emergency use authorisation (EUA) by the FDA are based on the RT‐PCR method and require proprietary instruments and long assay running times. Notably, increasing point‐of‐care (POC) RT‐PCR based tests are being developed, such as Cepheid and Biofire. These POC tests usually contain all the reagents in one cartridge and reduce the total testing time by using microfluidics and automation.

Isothermal amplification methods are a promising low‐cost alternative to replace traditional PCR devices. Bypassing the thermal cycle in the PCR process provides a faster amplification process, therefore, shorter sample‐to‐answer time. Using this technology, Abbott has developed ID NOW™ COVID‐19 assay that can provide a result within 5 min with a sensitivity of 0.125 copies/μL.

In the long run, we envisage the development of mixed technologies, combining molecular diagnostics and lateral‐flow assays. The Accula SARS‐CoV‐2 test by Mesa Biotech uses RT‐PCR technology to amplify the RNA signal of SARS‐CoV‐2 followed by lateral‐flow assays to reveal the result, thereby circumventing the need for fluorescence probes and detectors. CRISPR‐based diagnosis represents a prime example where mixed technologies can lead, combining high specificity and sensitivity with efficient and low‐cost biosensors. Direct quantification of viral RNA with CRISPR, without the need for DNA amplification, would reduce the sample‐to‐answer time significantly. Repurposing Sanger sequencers and Next Generation Sequencers for COVID‐19 diagnostics, not only opens the door to tracking the virus evolution, but it can also be a tool for high‐throughput testing.

The SARS‐CoV‐2 pandemic has caused an urgent need for rapid diagnostic testing and stirred up the biosensing sector. This quick development will continue, and some of the work in this review may change as more data on COVID‐19 becomes available and results are scrutinized. Accurate and widespread testing is mandatory to reduce the uncertainties associated with the number of infected cases, the case fatality rate and the effective reproduction number *R*. In a population‐level study, even accurate tests can give high levels of false positives if the true prevalence is low. The choice of target populations and how tests are performed are important considerations. Mass screening representative samples of the population and contact tracing can guide public health authorities adapting their nonpharmaceutical interventions both in time and space, focusing the containment effort in affected communities and loosening lockdowns. It will also enable a quick assessment of the extent of the epidemic by identifying asymptomatic cases and determining what fraction of the population has ever been infected and might have acquired immunity to COVID‐19.

## Conflict of interest

The author declares no conflict of interest.

## Biographical Information

*Ibon Santiago studied physics at the University of the Basque Country. He received an MSc from the Massachusetts Institute of Technology and a doctorate in physics from the University of Oxford, where he worked in DNA nanotechnology and active matter physics. He is currently Alexander von Humboldt Research Fellow at the Technical University of Munich. His research focuses on the physics of synthetic biological systems*.